# Evaluation of Mechanical and Elemental Properties of Bioceramic-Coated Orthodontic Brackets and Enamel Surface

**DOI:** 10.1055/s-0044-1789003

**Published:** 2024-09-18

**Authors:** Abdul Samad Khan, Ahlam AlAbdali, Nadia Irshad, Othoob AlMusayyab, Norah AlQahtani, Asma Tufail Shah, Sultan Akhtar, Yassine Slimani

**Affiliations:** 1 Department of Restorative Dental Sciences, College of Dentistry, Imam Abdulrahman Bin Faisal University, Dammam, Saudi Arabia; 2 College of Dentistry, Imam Abdulrahman Bin Faisal University, Dammam, Saudi Arabia; 3 Department of Dental Materials, Sharif Medical and Dental College, Lahore, Pakistan; 4 Interdisciplinary Research Centre in Biomedical Materials, COMSATS University Islamabad, Lahore Campus, Pakistan; 5 Department of Biophysics, Institute for Research and Medical Consultations, Imam Abdulrahman Bin Faisal University, Dammam, Saudi Arabia

**Keywords:** dental, white spots, orthodontic brackets, orthodontic adhesives, hydroxyapatite, bioactive glass, spin coating, shear strength

## Abstract

**Objective** The aim is to coat orthodontic brackets with two different bioactive materials and to compare the mechanical and morphological properties of coated brackets and tooth surfaces.

**Materials and Methods** A total of 120 stainless steel brackets were divided equally into three groups, that is, the uncoated brackets and nanohydroxyapatite (nHA)-coated, and nanobioactive glass (nBG)-coated brackets using a spin coater machine. The brackets were bonded on the enamel surface and underwent remineralization/demineralization cycles for days 1, 7, 14, and 30. At each time interval, the bond strength of the brackets was assessed using mechanical loading. An optical and scanning electron microscope (SEM) were used for surface evaluation, and the adhesive remanent index (ARI) values were obtained and quantified.

**Statistical Analysis** One-way analysis of variance using Tukey's test was used to compare the differences among the groups.

**Results** A uniform distribution of nanoparticles occurred on the surfaces of brackets. The shear bond strength (SBS) showed no significant differences in any tested groups on days 1, 7, and 14. However, control and nBG showed a significant difference from nHA at day 30. On days 7, 14, and 30, the nHA group showed the highest SBS values among the groups. For ARI, most samples showed an adhesive nature of failure at the enamel–brackets interface. The images confirmed the presence of coated particles on brackets and remnants of adhesives after SBS.

**Conclusion** This study confirmed that the nHA- and nBG-coated brackets have a high potential for application in orthodontics regarding structural and mechanical properties.

## Introduction

According to the World Health Organization (WHO), malocclusion is one of the most important oral health problems after caries and periodontal disease, with a prevalence rate of ∼39 and 93% in children and adolescents, respectively. 1 Global orthodontics reached $7.77 billion in 2022 and is expected to reach $26.35 billion by 2029. Orthodontic treatment aims to improve esthetics and function, 2 and researchers are putting all their efforts into constantly improving the materials related to orthodontic brackets and adhesive systems. Despite all the efforts in material improvements, ecological changes in the oral environment can initiate bacterial adhesion to the bracket–adhesive–enamel complex. 3

The plaque accumulation around brackets and acidic byproducts can result in enamel demineralization or the formation of white spots, subsequently leading to caries. 4 In this event, orthodontic treatment could harm the patients and reduce the treatment's successful outcome. 5 Orthodontic appliances are mainly made of stainless steel, which has less of an ability to reduce enamel demineralization. 6 The presence of plaque around the brackets can cause caries, gingivitis, and periodontitis. 7 Furthermore, in areas of enamel etching, the mechanical properties of the enamel surface also decrease after the bracket bonding using the etch and rinse adhesive system. This, in turn, can increase the risk of enamel microcracking during the debonding procedure. The orthodontic brackets' bond strength should be sufficient to withstand the forces applied during the treatment procedure. 8

The demineralization around the bracket bonded area should be prevented, and ideally, remineralization should be enhanced. One of the ways to enhance remineralization is by increasing the calcium and fluoride content in the oral fluids near the bonded brackets. 9 Bioceramics have been widely investigated with multiple applications in orthodontics, such as orthodontic wires, dental brackets, and dental bonding systems to improve mechanical properties and chemical corrosion. 1 10 The bioactive materials have a tendency to interact with the adjacent tissue and can form hydroxyapatite (HA) crystals, release ions, and ultimately establish a chemical bond between the material and tissue. 11 Among the bioactive materials, HA and bioactive glass (BG) have been studied in dentistry for the past two decades mainly in restorative dentistry. 12 HA is considered an osteoconductive and biocompatible material with better mechanical properties. 13 BG has osteoinductive behavior and can bond to soft and hard tissues, releasing remineralizing ions. Modifying the metallic orthodontic surface with bioactive materials can prevent demineralization of the enamel surrounding the brackets and promote remineralization after bracket debonding. 14

Recently, researchers have conducted different surface modification processes, such as coatings and surface treatments for orthodontic appliances, to render a material more suitable and less harmful. 15 ·Boccaccini et al 16 studied the use of BG coating on nitinol wires. Al Shehab et al 17 evaluated the effect of electrophoretic deposition of BG coating on orthodontic stainless steel wire and found significant acid-neutralizing capabilities and the ability to mitigate enamel demineralization. The use of coatings modifies the properties of various elements and changes their interactions with hard and soft tissues. 18 Various surface modification technologies such as laser cladding, electrophoretic deposition, sol-gel, and physical vapor deposition have been developed and employed to coat surfaces. 19 20 These physical methods of coatings can alter the structure on the micro- and nanoscale. 21 Unfortunately, some of these technologies show drawbacks, such as poor bonding strength between surface and coatings, the induction of phase transformation, time-consuming, and difficulty in depositing a uniform layer on complex-shaped workpieces. 22

Spin coating is a unique and reliable technique that produces a thin, uniform coating film. The viscous force and surface tension cause a thin residual film to be retained on the flat substrate. The advantages of the spin coating technique are that it can achieve tailored thickness and uniform coating; moreover, this technique is cost-effective and has a fast operating process. 23 Surface modifications with materials having bioactive effects aid in better therapeutic results and reduce the risk of complications. The authors could not find any study where the inner surface of the brackets was coated with the HA and BG materials. Therefore, the present study aimed to coat orthodontic brackets with two different bioactive materials using the spin coating technique and compare the mechanical and morphological properties. It was hypothesized (H _1_ ) that the bioactive materials coated brackets would show improved strength and reduce the demineralizing effect on the tooth surface. The alternative hypotheses were pitted against the null hypothesis (H _0_ ).

## Materials and Methods

Commercial stainless steel premolar brackets (LOT # 431240-48; discovery smart, McLaughlin 22, Dentaurum, Ispringen, Germany) were used in this study. BG and HA nanoparticles were synthesized using sol-gel and microwave irradiation methods, respectively. Our group previously 24 25 reported the methods of preparation. The X-ray diffraction patterns of both particles are given in Supplementary Fig. S1 (available in the online version only). For the BG group, the diffractogram showed an amorphous pattern with a low-intensity peak at 2ϑ value ∼ 29.12 (d-spacing 2.99 Å), which could be due to calcium carbonate (Joint Committee on Powder Diffraction Standards (JCPDS) data file no. 001-0482). For the HA group, the crystallographic characteristic peaks were identified using JCPDS data file no. 46-0905. The obtained particle size of BG and HA was 92 and 20 to 50 nm, respectively.

**Fig. 1:**
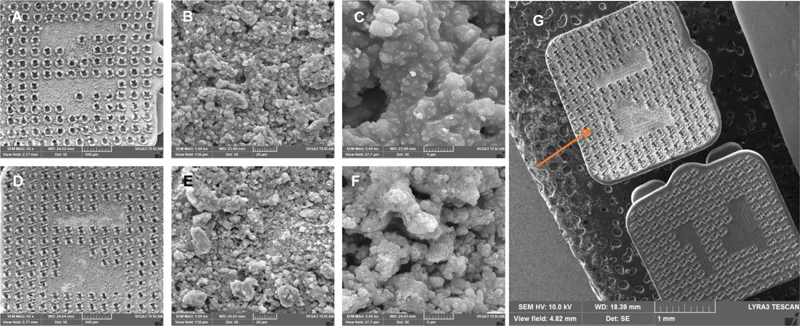
SEM images showing the coating of ( **A** – **C** ) nHA and ( **D** – **F** ) nBG on bracket surfaces. The images show the uniform distribution of nanoparticles. The nanosize is visible at higher magnification, ( **G** ) comparative image of coating (with arrow) and noncoated bracket. nBG, nanobioactive glass; nHA, nanohydroxyapatite; SEM, scanning electron microscope.

For preparation of the coating solutions, 15.0 volume% polyethylene glycol (PEG; Sigma Aldrich, St. Louis, Missouri, United States) was prepared in deionized water at 80°C on a magnetic stirrer for 15 minutes. Then, the as-obtained nano-BG (nBG) (4.0 weight%) and nano-HA (nHA) (2.5 weight%) powders were mixed with PEG using a magnetic stirrer for 24 hours at room temperature. The weight concentration of both powders was optimized based on the variations in density and volume.

Coating of the brackets was done using the spin coating technique (WS-650 Spin Coater, Laurell Technologies, Corporation, Lansdale, Pennsylvania, United States), where five drops of mixed solutions were spun on each bracket at 2,500 rpm under a vacuum of 50 psi of nitrogen gas. The spin coating process was done in four cycles. Then, the brackets were placed in a drying chamber (ED 115, BINDER GmbH, Tuttlingen, Deutschland) at 120°C for 2 hours.

### Preparation of Teeth

After obtaining ethical permission from the Institutional Review Board, human-extracted caries-free teeth (premolars) were collected from the oral and maxilla-facial department of the institutional hospital. The teeth were extracted for orthodontic treatment. The obtained teeth were disinfected with 70/30 ethanol solution for 10 minutes and then stored in 0.1% thymol solution at a refrigerated temperature. The teeth were embedded in self-cured acrylic resin in such a way that crowns were left exposed by 2 mm below the cementoenamel junction.

Before the bonding procedure, the labial surface of all teeth was submitted to a rubber cup prophylaxis using extrafine pumice and water slurry for 15 seconds. Then, the specimens were thoroughly washed with air/water spray for 10 seconds and air-dried. The teeth were randomly assigned to three groups based on noncoated and coated orthodontic brackets.

The sample size was calculated per the WHO's specifications, keeping the power of study equal to 90% and the significance level equal to 5%. 26 The mean and standard deviation values were obtained from a previous study. 27 The calculated sample size was 27; however, it was increased to 50 per group.

A total of 150 stainless steel premolar brackets were randomly divided into three groups based on noncoated (control group) and coated orthodontic brackets (nHA-coated and nBG-coated). The labial surface of each tooth crown was bonded with respective noncoated or coated stainless steel brackets using standard bonding protocols; the tooth surface was etched with 37% phosphoric acid gel (FineEtch37, Incheon, Korea) for 15 seconds and then rinsed thoroughly with water. The tooth surface was air-dried gently, and a thin layer of primer was applied. After that, the adhesive was applied onto the mesh pad of each bracket (Transbond-XT, 3M Unitek, Monrovia, California, United States). The brackets were placed on the tooth surface, and excessive adhesive was removed with a sharp scaler. The adhesives were light cured using high-intensity light-emitted diode light (power: 1,200 mW/cm ^2^ , wavelength; 470 nm; Woodpecker, China) for 20 seconds each from the mesial and distal sides to complete the process of bonding. It was maintained that an equal amount of adhesives was placed on each bracket surface.

### pH Cycling

According to the pH cycling protocol, each tooth crown was immersed in 4 mL of demineralizing solution for 8 hours and kept in an incubator at 37°C. After that, the specimens were removed and rinsed thoroughly with deionized water to wash out excess demineralizing solution, then immersed in 4 mL of remineralizing solution in the same environment for the next 16 hours. The demineralization and remineralization solutions were prepared as described previously. 28 A fresh solution was prepared after every third day. This cycle was repeated for days 1, 7, 14, and 30.

### Shear Bond Strength Test

The shear bond strength (SBS) of noncoated and coated bracket groups ( *n* = l0) was assessed at each interval, that is, days 1, 7, 14, and 30. At each specific period, the samples were removed from the solution and were washed with deionized water, and incubated at 37°C for 2 hours. Then, the brackets were removed using mechanical loading (ElectroPuls E3000, Instron, United States) by a stainless steel jig (tip size = 1.5 mm) with a crosshead speed of 0.5 mm/min and load cell was 1 kN. The surface area of the brackets was calculated, and the bond strength values were obtained in MPa based on the obtained surface area and force.

### Morphological and Elemental Analysis

An optical microscope (Luxo, Elmsford, New York, United States) at ×10 and ×40 was used to assess the remaining adhesive on the tooth and bracket surfaces. The adhesive remanent index (ARI) and the mode of bond failure were investigated. ARI was quantified according to the criteria established by Årtun and Bergland, 29 where 0 = no adhesive left on the tooth; 1 = less than half of the adhesive left on the tooth; 2 = more than half of the adhesive left on the tooth; and 3 = all the adhesives left on the tooth.

The morphological analyses of the teeth and brackets surfaces were conducted using scanning electron microscopy/energy dispersive spectroscopy (SEM/EDS; VEGA-3 LMU; Tescan, Czech Republic) at 15 kV. Before SEM analysis, the samples were gold coated (Quorum Technologies, Lewes, UK), and then the images were taken at various magnifications to record different features of the specimens.

### Statistical Analysis

The mean values of SBS were analyzed statistically by one-way analysis of variance post hoc Tukey's and Kruskal–Wallis' tests for multiple comparisons using the SPSS version 22 (IBM Software, Chicago, Illinois, United States). Mann–Whitney's *U* test and *t* -test were also used to compare the two groups. Frequencies of ARI scores were obtained, whereby the significance level was set at 5%.

## Results

A uniform distribution of coating materials was achieved after the spin coating process. The brackets were coated with nHA and nBG nanomaterials, and the quality of the coating was evaluated by SEM. Fig. 1A to C ) and Fig. 1D to F show the SEM images of nHA and nBG coatings, respectively, on brackets at different magnifications. The SEM images showed the average particle diameter of ∼ 50 to 200 nm for nHA and nBG on the coated bracket surface. Fig. 1G shows the comparative image of the coated and noncoated brackets, where the coated bracket showed bright contrast due to the presence of coating material.

### Shear Bond Strength

**Fig. 2:**
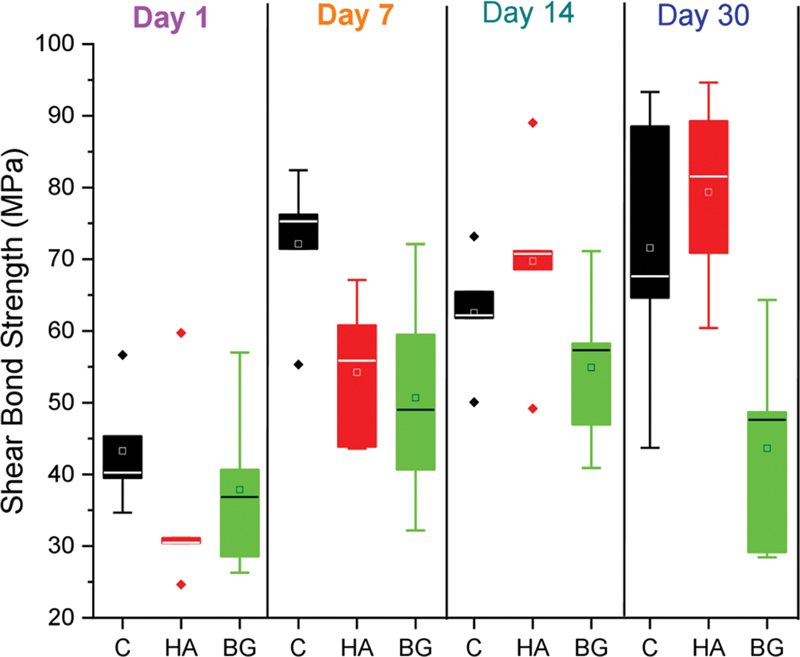
Graph showing the mean and standard deviation of shear bond strength values (MPa) of C and nHA-coated and nBG-coated brackets at days 1, 7, 14, and 30. A nonsignificant difference was observed except at day 30, where a significant difference was found; control versus nBG-coated and nHA-coated versus nBG-coated. C, control; nBG, nanobioactive glass; nHA, nanohydroxyapatite.

The comparative means and standard deviation bond strength values are shown in Fig. 2 and Table 1 . It was observed that on day 1, the control group showed the highest SBS value (43.26 ± 8.37 MPa) compared with nHA-coated (35.27 ± 13.91 MPa) and nBG-coated (37.87 ± 12.19 MPa) groups, and a similar trend was observed at day 7, where the control group showed the highest SBS (72.14 ± 10.19 MPa) compared with nHA-coated (54.23 ± 10.39 MPa) and nBG-coated (50.68 ± 15.66 MPa) groups. In contrast, on days 14 and 30, the nHA-coated group showed the highest SBS values, followed by the control and nBG-coated groups. For the control and nBG-coated groups, a nonlinear behavior was observed, where the control group showed an increase in value till day 7, then it was reduced at day 14 and then increased at day 30, whereas for nBG-coated group, the values were increased till day 14, then its decreased. However, the nHA-coated group showed a steady increase in values with time.

**Table 1:** The mean shear bond strength and standard deviation values of the C, nano-HA, and nano-BG groups at days 1, 7, 14, and 30

	C	HA	BG	*p* -Value
Day 1	43.26 ± 8.37	35.27 ± 13.91	37.87 ± 12.19 a	0.286
Day 7	72.14 ± 10.19 b c	54.23 ± 10.39 b	50.68 ± 15.66	0.060
Day 14	62.54 ± 8.33 b d	69.72 ± 14.13 ^b.d^	54.91 ± 11.61 a	0.060
Day 30	71.56 ± 20.00 c d	79.34 ± 13.84 d	43.63 ± 15.08	0.001
*p* -Value	0.007	0.0004	0.251	

Abbreviations: BG, bioactive glass; C, control; HA, hydroxyapatite.

a Significant difference between days 1 and 14.

b Nonsignificant difference between days 7 and 14.

c Nonsignificant difference between days 7 and 30.

d Nonsignificant difference between days 14 and 30.

At each time interval, nonsignificant difference ( *p* < 0.05) was found within the groups, except at day 30, where a significant difference ( *p* = 0.001) was observed. Statistically, a nonsignificant difference ( *p* < 0.05) was observed between the control and nHA-coated groups at each time interval, and a similar trend was observed between the control and nBG-coated groups; however, at day 30, a significant difference ( *p* = 0.001) was observed. No statistically significant differences ( *p* < 0.05) were observed between the nHA-coated and nBG-coated groups at each time interval, except at day 30, where a significant difference ( *p* = 0.003) was observed among all the groups at different time intervals ( *p* < 0.05).

A significant difference ( *p* = 0.007) was observed within the control group at each time interval; however, a nonsignificant difference was found between days 7 and 14 ( *p* = 0.141), days 7 and 30 ( *p* = 0.955), and days 14 and 30 ( *p* = 0.378). For the HA group, a significant difference ( *p* = 0.0004) was observed within the group at each time interval; however, a nonsignificant difference was found between days 7 and 14 ( *p* = 0.083) and days 14 and 30 ( *p* = 0.308). A nonsignificant difference ( *p* = 0.251) was observed within the BG group at each time interval, whereas a significant difference ( *p* = 0.05) was found between days 1 and 14.

### Adhesive Remnant Index

**Fig. 3:**
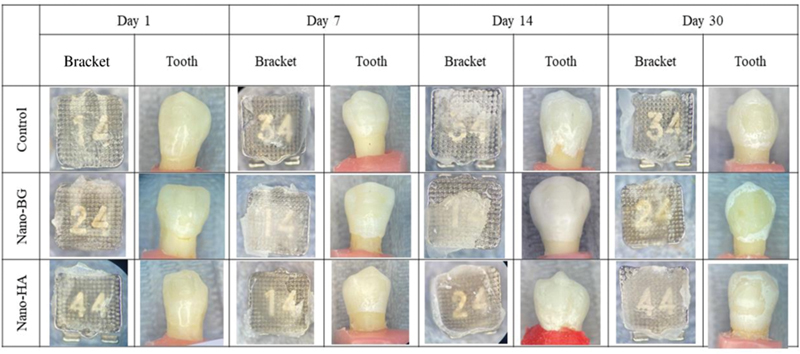
Optical microscopic pictures of the brackets and teeth surface after debonding at days 1, 7, 14, and 30. BG, bioactive glass; HA, hydroxyapatite.

The optical microscope analysis after debonding did not reveal any fractures or cracks on the enamel surfaces. The ARI was quantified by means of a dental explorer and optical microscope ( Fig. 3 ). Evaluation of the ARI showed no statistically significant differences in the distribution of scores between the groups ( Table 2 ). Most of the samples had an ARI score between 0 and 1 with an adhesive nature of failure at the enamel/brackets interface. The control group showed a score of 1 for 60 to 70% of samples; the nBG group showed a 70 to 80% score of 1, and the nHA group showed 50 to 70%. However, on day 14, only 10% of samples of the nHA group showed a score of 1.

**Table 2:** ARI scoring (%) distribution of the control and coated brackets on days 1, 7, 14, and 30

Days/scoring/groups	Day 1				Day 7				Day 14				Day 30			
	0	1	2	3	0	1	2	3	0	1	2	3	0	1	2	3
Control	30	60	10	0	40	60	0	0	20	70	10	0	10	60	20	10
nBG	10	80	10	0	30	60	10	0	30	70	0	0	20	50	20	10
nHA	40	50	10	0	30	70	0	0	60	10	0	20	20	50	20	10

Abbreviations: ARI, adhesive remanent index; nBG, nanobioactive glass; nHA, nanohydroxyapatite.

**Fig. 4:**
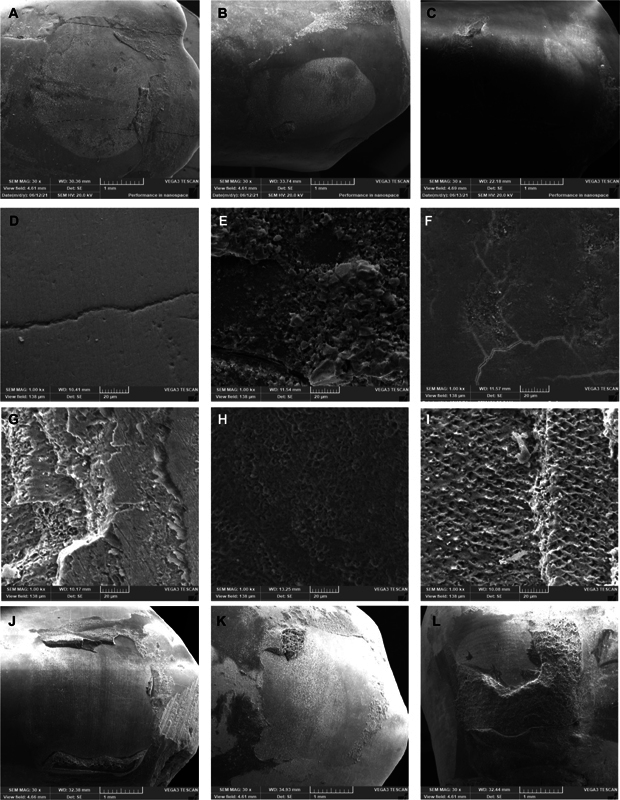
SEM images of teeth surface after debonding of the control, nHA-coated, and nBG-coated brackets at ( **A** – **C** ) day 1, ( **D** – **F** ) day 7, ( **G** – **I** ) day 14, and ( **J** – **L** ) day 30, respectively. nBG, nanobioactive glass; nHA, nanohydroxyapatite; SEM, scanning electron microscope.

**Fig. 5:**
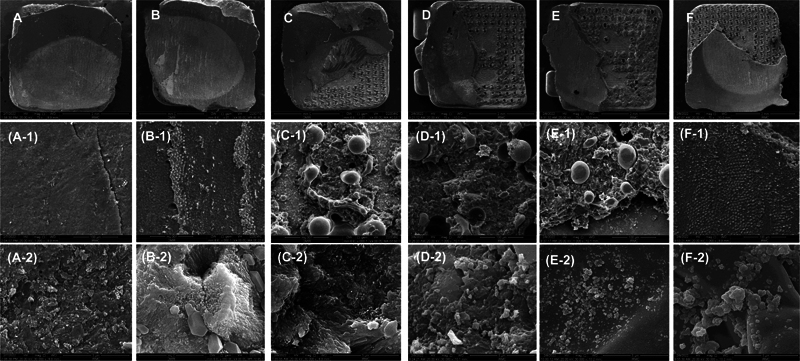
SEM images of brackets after debonding at days 7 and 30, where ( **A** , A-1, A-2) control group at day 7 and ( **D** , D-1, D-2) day 30, nHA-coated at ( **B** , B-1, B-2) day 7 and ( **E** , E-1, E-2) day 30, nBG-coated at ( **C** , C-1, C-2) day 7 and ( **F** , F-1, F-2) day 30. nBG, nanobioactive glass; nHA, nanohydroxyapatite; SEM, scanning electron microscope.

After debonding, both teeth and brackets were analyzed by SEM to study the failure mode ( Figs. 4 and 5 ). The represented micrographs of the teeth surface from where the brackets were debonded are shown in Fig. 5 , where Fig. 4A, D, G, J is control teeth (left panel), while Fig. 4B, E, H, K and Fig. 4C, F, I, L represented the nHA- and nBG-coated teeth, respectively, after 1, 7, 14, and 30 days. The SEM images displayed the mode of adhesive failure, and it was mostly adhesive behavior at the enamel–adhesive interface for all groups except the nHA-coated group, where some of the samples showed a mixed (adhesive and cohesive) behavior. The nBG group also showed mixed behavior on day 30 ( Fig. 4L ). In addition, no damage was detected on the surface of the teeth.

After debonding on day 7 ( Fig. 5A–C ) and day 30 ( Fig. 5D–F ), the representative SEM images of brackets are presented at different magnifications. The debonded brackets on day 7 show almost complete coverage of adhesive on the bracket surface for the control and nHA-coated brackets, whereas partial coverage was observed for the nBG-coated brackets. However, on day 30, the adhesive was partially present on the debonded brackets. At higher magnification, the presence of nanoparticles and layering features are visible ( Fig. 5E -2 , F-2 ).

**Fig. 6:**
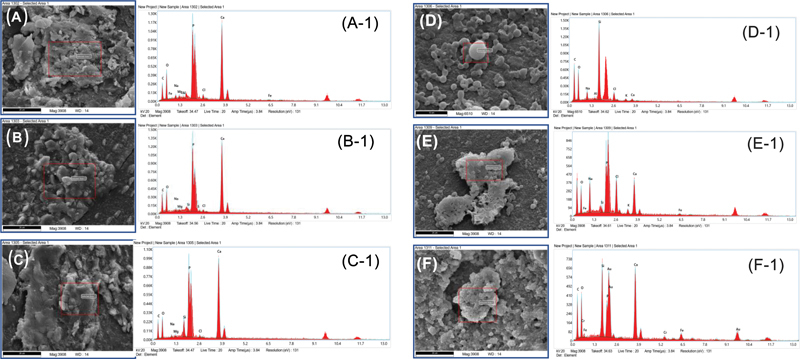
EDS spectra along with corresponding images of the control group ( **A** , A-1) at day 7 and ( **D** , D-1) at day 30; nHA-coated ( **B** , B-1) at day 7 and ( **E** , E-1) at day 30; nBG-coated ( **C** , C-1) at day 7 and ( **F** , F-1) at day 30. The Ca and P peaks are visible for all specimens; however, the Si peak is only visible for the nBG-coated group. EDS, energy dispersive spectroscopy; nBG, nanobioactive glass; nHA, nanohydroxyapatite.

The EDS spectra, along with corresponding images of teeth surfaces after debonding at days 7 and 30 of the control group, are shown in Fig. 6A, A-1, D, D-1) , nHA-coated ( Fig. 6B, B-1, E, E-1 ), and nBG-coated ( Fig. 6C, C-1, F, F-1 ) confirmed the presence of Ca and P elements. The Ca/P ratio (nHA-coated specimens) at days 7 and 30 was 2.2 and 1.7, respectively, whereas the nBG-coated group showed Ca/P ∼ 2.2 at day 30. Moreover, the presence of Si is apparent in the nBG-coated group confirmed the presence of BG on the surface. The nHA and nBG groups showed a newly formed layer with the presence of trace amounts of silica at day 30.

## Discussion

Orthodontic treatment is necessary to address esthetic and functional issues and contributes to developing white spot lesions (WSLs). 30 Meticulous attention is required to maintain a high quality of oral hygiene, which can help avoid enamel demineralization. Demineralization can frequently happen throughout a lengthy orthodontic treatment course. An acidic pH of around 5.5 promotes demineralization. When this equilibrium is shifted in favor of remineralization, WSL may shrink or vanish entirely. 31

The present results showed that in terms of bond strength, the coating of brackets with nHA and nBG had no statistically significant difference initially; however, on day 30, a significant difference was observed, and the nBG group showed low values. However, the low values are still within the acceptable range. No clear guidelines regarding the shear force limit for orthodontic bracket placement have been specified in the literature. The only prerequisite for an orthodontic biomaterial is that it should allow good adhesion to bear the masticatory and other functional forces applied during the treatment (minimum bond strength of 6–10 MPa). 32 In the debonding process, strong adhesive forces are not required to avoid enamel loss. Although these limits are mostly theoretical, an ideal orthodontic biomaterial should have bonding forces in the interval of 5 to 50 MPa. The results of the SBS data of the various groups in this study fall within the theoretical limits. In orthodontic treatment, bracket adhesion transmits specific forces to the teeth, which are important for the end results of the treatment. If the bond strength is increased, the chances of bracket debonding are decreased, which, in turn, has the added advantage of saving time and preserving the healthy enamel surface, as constant debonding of the brackets increases the treatment time, cost, and can compromise the integrity of the enamel. 33

It is preferable to have low ARI scores (failure within the adhesive layer) when the brackets are separated from the tooth on completion of the treatment period to lessen the possibility of the enamel surface breaking down. 34 The current study had ARI scores that were mostly 0 or 1, which matched the research findings published previously. 35 In this study, a high SBS value is seen in the control and nHA-coated groups, whereas the SBS value was low in the nBG-coated group; however, the percentage value of the ARI in nBG-coated group was high compared with the control and nHA groups although not significantly. The findings of this study are in agreement with the findings of the previous study, 36 which states that the ARI is inversely proportional to the SBS. The decrease in the SBS value in the nBG-coated group could be due to the quick release of calcium, phosphate, and silicon ions compared with the nHA-coated group. This prevents the establishment of bonds between enamel and methacrylate acidic monomers in a competitive manner and affects the bond strength in this area. 37 However, the release of ions can help in apatite formation.

Clinically, the orthodontic adhesives are mechanically in contact with the tooth surface, which can also act as a barrier between released ions and the tissue surface. Nevertheless, calcium and phosphate ions released from the bracket surface can also reach the surface of teeth, rebuilding the demineralized structure. The findings observed in this study are in accordance with the literature, where it is reported that the ions can diffuse from the adhesive layers in an acidic environment. 38 In this study, the pH cycle was extended to 30 days; however, it was still unable to address the complex process occurring in the clinical situation. The release of ions is in correlation to the solubility of the HA and BG, whereby continuous contact with the solvent enhances the ion release behavior. 39 It is expected that the supersaturated level of calcium and phosphate ions diffused deep toward the demineralized surface, thereby increasing the mineral content of the enamel, which is evident from the EDS data. The findings are in accordance with the previously reported literature. 40 41

BGs typically exhibit distinct biological behaviors depending on the surroundings in which they are housed. 42 Compared with HA, BGs have superior bioactivity, degradability, osteoinductive properties, and reactivity, most likely as a result of the biologically active, silica-rich layer that is created when ions leach from the BG structure, which significantly stimulates the development of a new apatite layer. 43 It has been shown that adding BG nanoparticles to orthodontic adhesive cements produced remineralizing effects and is more efficient compared with HA. 44

nHA-coated and nBG-coated brackets displayed the appearance of spherical agglomerates. Additionally, a sizable volume of crystals was seen to develop in the nHA-coated group, and distinct precipitate deposition was visible. The element concentration measured by EDS analysis revealed that the nHA-coated group had a considerably greater calcium and phosphate concentration than the nBG-coated group at day 30, whereas at day 7, nBG-coated group showed a higher concentration of both. In this study, the used nHA particles were heat treated at 800°C. The low crystallinity of nHA contributed to the release of Ca and P. Increased saturation of oral fluids with HA would favor the deposition of apatite minerals in the lesions, ultimately encouraging remineralization. In the nBG-coated group, the reduction in the calcium and phosphate ion release over time is in accordance with the study conducted by Kim et al. 45 It was peculiar that calcium levels also dropped, even though the fall in phosphate levels was probably due to the deposition of calcium phosphate. The calcium ion releases more than the phosphate ions, and quantitatively, calcium was shown to be ∼20 times more effective than phosphate in preventing enamel demineralization. 46 In this study, the nanosized particles with high surface area were used to coat the bracket surface. The size of the particles directly impacts the surface-to-volume ratio and it is evident that the higher surface area to volume could favor the release of ions. The change in pH behavior can influence the ion release pattern, where the calcium and phosphate ions release more at lower pH (4–5.5). 47 It is reported that after sugar intake, the *in vivo* pH drops below the critical value (pH = 5.5). 48 Therefore, it is expected that in such conditions, the release of ions can minimize the risk of demineralization. This could be due to the classic ion nucleation theory, where continuous deposition of ions can facilitate apatite formation. It is assumed that the coating of the bioactive particles on the bracket surface could enhance the physical properties without compromising the mechanical properties. The current study showed that acceptable bond strength and the ion release pattern can minimize the risk of demineralization, which is mainly associated with orthodontic treatment. The literature supports that the enamel surface is compromised during the orthodontic treatment and after the debonding 49 50 ; therefore, the additional coating of the bioactive materials would be expected to improve the clinical outcomes.

This study investigated the bond strength behavior under the influence of pH challenge and simulated the clinical environment. Certain factors limit this study in comparison to the extent of the surface area of remineralization between the HA and BG groups, and its effect on SBS needs to be evaluated. Moreover, in clinical situations, various forces are exerted from different directions on the brackets, and these cannot be generalized in *in vitro* bond strength studies. In this study, the coating was performed only on metal brackets; however, it is recommended that coating should be performed on ceramic brackets in future studies and investigate the bond strength.

## Conclusion

Within the limitation of this study, it is concluded that the null hypothesis is rejected. In relation to the SBS, all groups presented similar results, except for the nBG-coated group at day 30, which showed a statistically significant difference. Control and nHA-coated groups yielded the highest SBS value. Adding BG nanoparticles might reduce SBS; however, the adhesion might still be acceptable. The coated brackets presented more bond failures at the enamel/adhesive interface. Although this interface is considered dangerous because of the risk of damaging the enamel surface, no damage was observed to teeth after debonding.
