# Supplementary figures and images for: Evaluation of Mechanical and Elemental Properties of Bioceramic-Coated Orthodontic Brackets and Enamel Surface
Source: Eur J Dent. 2024 Sep 18;19(2):389–98. doi: 10.1055/s-0044-1789003 (PMC12020588; doi:10.1055/s-0044-1789003)

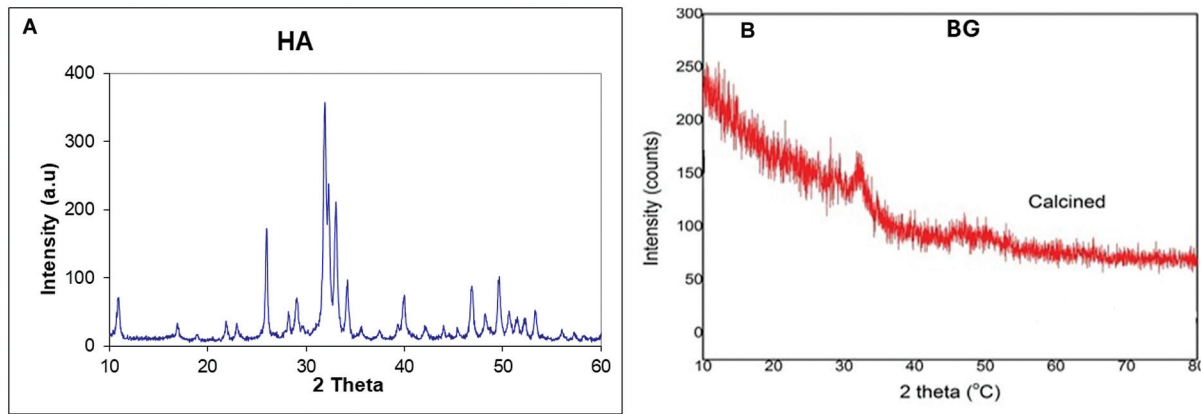

Supplementary Fig. S1 X-ray diffractogram of (A) hydroxyapatite (HA) and (B) bioactive glass (BG).

Supplement: Supplementary file 1 — Supplementary Material [file 10-1055-s-0044-1789003-s2433453.pdf]
